# Correlation of the absorbed dose to the blood and DNA damage in leukocytes after internal ex-vivo irradiation of blood samples with Ra-224

**DOI:** 10.1186/s13550-018-0422-4

**Published:** 2018-08-06

**Authors:** Sarah Schumann, Uta Eberlein, Jessica Müller, Harry Scherthan, Michael Lassmann

**Affiliations:** 1 Department of Nuclear Medicine, University of Würzburg, Oberdürrbacher Str. 6, 97080 Würzburg, Germany; 2 Bundeswehr Institute of Radiobiology affiliated to the University of Ulm, Neuherbergstr. 11, 80937 Munich, Germany

**Keywords:** DNA damage, γ-H2AX, 53BP1, Biological dosimetry, Absorbed dose to the blood, α-Emitter, Ra-224

## Abstract

**Background:**

Irradiation with α-particles creates densely packed damage tracks along particle trajectories in exposed cells, including complex DNA damage and closely spaced double-strand breaks (DSBs) in hit nuclei. Here, we investigated the correlation of the absorbed dose to the blood and the number of α-induced DNA damage tracks elicited in human blood leukocytes after ex-vivo in-solution exposure with Ra-224. The aim was to compare the data to previously published data on Ra-223 and to investigate differences in DNA damage induction between the two radium isotopes.

**Results:**

Blood samples from three healthy volunteers were exposed ex-vivo to six different concentrations of Ra-224 dichloride. Absorbed doses to the blood were calculated assuming local energy deposition of all α- and β-particles of the Ra-224 decay chain, ranging from 0 to 127 mGy. γ-H2AX + 53BP1 DNA damage co-staining and analysis was performed on ethanol-fixed leukocytes isolated from the irradiated blood samples. For damage quantification, α-induced DNA damage tracks and small γ-H2AX + 53BP1 DSB foci were enumerated in the exposed leukocytes. This revealed a linear relationship between the frequency of α-induced γ-H2AX damage tracks and the absorbed dose to the blood, while the frequency of small γ-H2AX + 53BP1 DSB foci indicative of β-irradiation was similar to baseline values.

**Conclusions:**

Our data provide a first estimation of the DNA damage induced by Ra-224 in peripheral blood mononuclear cells. A comparison with our previously published Ra-223 data suggests that there is no difference in the induction of radiation-induced DNA damage between the two radium isotopes due to their similar decay properties.

## Background

Between the mid-1940s and the late 1990s, Ra-224 dichloride was particularly used in Germany for treating different bone and joint diseases [1]. The first application was the use of Ra-224 dichloride in a mixture with platinum and eosin for treating children and juveniles suffering from bone tuberculosis. Later on, pure Ra-224 dichloride was administered mainly for pain palliation therapy of ankylosing spondylitis (AS) patients [1]. The total activity given within the therapy period differed between the cohorts analyzed (1945–1955: 0.66 MBq∙kg^−1^ [2] and 1948–1975: 0.17 MBq∙kg^−1^ [3]). The production of Ra-224 dichloride was stopped in 1990 because of technical and commercial reasons [3]. Between 2000 and 2005, Ra-224 dichloride was again available in Germany for bone pain palliation in AS patients, but the administered activity was reduced to a total activity of 10 MBq from ten injections of 1 MBq each per week [1]. Due to the enhanced risk of malignant disease following the injections of Ra-224 dichloride, the clinical use of this nuclide for treating AS patients was discontinued in 2005 [3]. Most recently, Westrom et al. investigated the use of Ra-224-coated microparticles for localized internal α-therapy in mice [4, 5]. Furthermore, Juzeniene et al. showed in a mouse model that Ra-224 is a promising candidate for the treatment of bone metastases related to breast cancer [6]. These publications highlight the renewed interest in using Ra-224 for clinical applications.

In 2013, another radium isotope, the α-emitter Ra-223, received marketing authorization in Europe for treating castration-resistant prostate cancer patients with widespread bone metastatic disease [7], leading to the treatment of numerous patients with this radiopharmaceutical. Moreover, there is growing interest to use other α-emitters for targeted radionuclide therapy. Examples of targeted cancer therapy are discussed by Sgouros et al. [8]. Such applications include, but are not limited to, the treatment of melanoma or leukemia with Bi-213-labeled antibodies or ovarian carcinoma with At-211-labeled antibodies [9]. The most recent examples are the treatment of metastatic prostate cancer with Ac-225- or Bi-213-PSMA ligands [10, 11] and radioimmunotherapy with Pb-212-TCMC-Trastuzumab [9, 12].

The absorbed dose to the blood is frequently used as a surrogate marker for the absorbed dose to the bone marrow as the hematopoietic system is a potential organ-at-risk for targeted radionuclide therapies [13]. Hence, it is important to learn about the radiation-induced DNA damage in blood leukocytes induced by internal irradiation with α-emitters and to determine the absorbed dose to the blood/DNA damage relationship. α-particles cause, in contrast to γ-or β-radiation, complex DNA damage and DSBs that are closely spaced along particle tracks and thus more difficult to repair [14, 15]. DNA damage induced by α-particles can be visualized in exposed nuclei as long tracks of γ-H2AX-positive chromatin and repair-associated proteins when the ionization tracks in the nuclei lie parallel to the focal plane of the observer [16–19].

So far, only a few publications studied the biological effects of α-emitters in human leukocytes and related these to the absorbed dose to the blood [19–21]. Stephan et al. studied chromosomal aberrations in peripheral lymphocytes of AS patients treated with Ra-224 dichloride in order to better understand the biological effect of Ra-224 to the hematopoietic system [20]. Recently, our group studied the DNA damage elicited in leukocytes by internal ex-vivo irradiation of blood with Ra-223 [19].

The aim of this study was to correlate the absorbed dose to the blood with the induced DNA damage in leukocytes after internal ex-vivo irradiation of blood with Ra-224 dichloride. It was motivated by the similar chemical and decay properties of Ra-223 and Ra-224, the increasing numbers of radionuclide therapies with α-emitters, and the lack of data on DNA damage induced by Ra-224 and its progeny. Moreover, with respect to historical risk data after the use of Ra-224 [2, 3], the comparison of the results of this study to the previously observed DNA damage data obtained by in-solution exposure with Ra-223 dichloride [19] might also contribute to an improved understanding of the differences and similarities between Ra-224 and Ra-223.

## Methods

### Specifications of Ra-224 and calculation of the absorbed dose to the blood

**Fig. 1:**
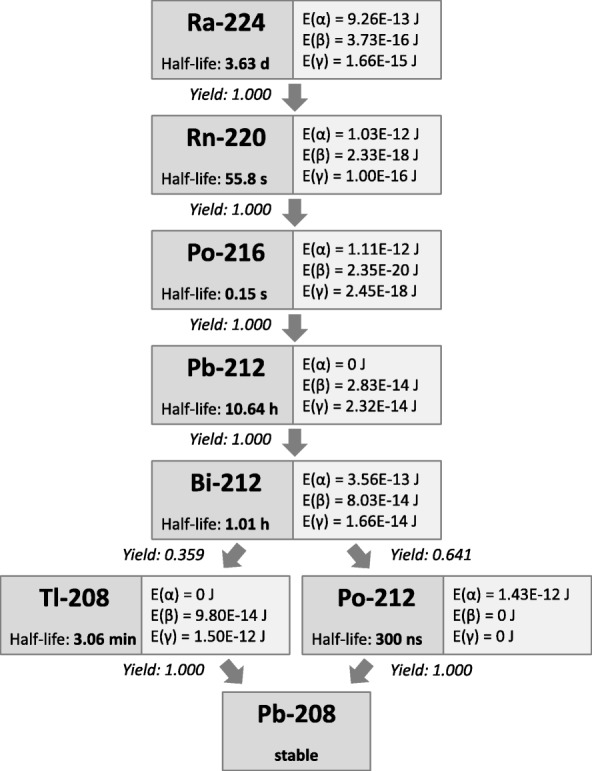
Decay chain of Ra-224 with data on half-lives [23] and energy deposition per transition [22]. E(α) and E(β) state the energy per transition for the α-particles and recoil nuclei (α) and the β-particles, conversion electrons and Auger electrons (β) considered for the calculation of the absorbed doses to the blood. E(γ) states the energy per transition for the X- and γ-radiation. However, E(γ) was not considered for the calculation of the absorbed doses to the blood due to the low interaction probability in the geometry of the incubation tube

Ra-224 is an α-emitter with a half-life of 3.631 days. As shown in Fig. 1, it decays in six steps into the stable nuclide Pb-208. Four of its progeny, Rn-220, Po-216, Bi-212, and Po-212, are also α-emitters. In total, four α-particles are emitted per decay, emitting energies between 5.30 MeV (Bi-212) and 10.55 MeV (Po-212) [22, 23]*.*

To calculate the absorbed doses to the blood, local energy deposition of all α- and β-particles of all progeny of Ra-224 was assumed. The contribution of the γ-emission was neglected due to the low interaction probability in the small sample volume. Based on these assumptions, absorbed dose coefficients (in mGy∙kBq^− 1^) for 1 h irradiation were determined for both the α- and the β-contribution to the absorbed dose to the blood.

Ra-224 dichloride was provided by ABX-CRO advanced pharmaceutical services (Dresden, Germany). The radiochemical purity of the product is > 99.99%. The contents of Th-228 are supposed to be less than 1 ppm. This has also been confirmed by a spectroscopic measurement of one of the samples 4 weeks after receiving the vial. No impurity could be detected.

### Blood sampling, irradiation, immunofluorescent staining, and evaluation of DNA damage

Blood samples were drawn from three healthy test persons (TP1: f, 34 years; TP2: m, 60 years; TP3: f, 22 years) using Li-Heparin blood collecting tubes (S-Monovette®; Sarstedt). In order to compare the results to our previous Ra-223 study [19], we recruited the same test persons. Each blood sample was split into six 3.5 ml aliquots. One non-irradiated aliquot per test person was prepared to determine the individual baseline value. The other five aliquots were supplemented with 1 ml of Ra-224 solution diluted with phosphate-buffered saline (PBS) to result in different activity concentrations. The radioactive blood samples were incubated for 1 h at 37 °C in a 5 ml round bottom tube. The processing of the blood samples, the immunofluorescent staining with γ-H2AX and 53BP1 antibodies, and the evaluation of the DNA damage followed the protocol described in [19].

DNA damage was categorized into two classes, (I) distinct small round foci typically seen in β-irradiated mononuclear blood cells [24] and (II) cells containing tracks of γ-H2AX and 53BP1 and cells with huge foci (Ø > 1.1 μm) likely resulting from α-hits laying perpendicular to the observed plane [19].

### Activity quantification

1 ml of the radioactive blood solution of each sample was measured in a calibrated, high purity germanium detector (Canberra). For activity quantification, emission lines of Ra-224 at 241.0 keV (emission probability of 4.12%) and its progeny Pb-212 at 238.6 keV (43.6%) and at 300.1 keV (3.18%) and Bi-212 at 727.3 keV (6.65%) were evaluated. The measurements were decay-corrected to the start time of the measurement. The mean activity value of the evaluated emission lines was used for the calculation of the absorbed doses.

### Statistical analysis

For data analysis and statistical evaluation, Origin (OriginPro 2017, Origin Lab Corporation) was used. To test whether data were distributed normally, the Shapiro-Wilk test was conducted. Results were considered as statistically significant at *p* < 0.05.

For the average number of α-tracks and small foci per cell, the standard deviation of each value was calculated assuming a Poisson-distribution.

For the α-contribution to the absorbed doses to the blood, a propagation of uncertainties was performed assuming that the activity, the time between the addition of the radioactive solution and the measurement in the germanium detector, the measuring volume, and the absorbed dose coefficient are independent variables with uncertainties.

In order to perform linear fits, the standard deviations of the counting process and the uncertainties of the calculation of the absorbed doses were considered. The corresponding correlation coefficients (Pearson’s *r*) are given.

## Results

### Absorbed dose calculation and activity quantification

Summing up all contributions, we obtained an absorbed dose coefficient of 16.1 mGy∙kBq^−1^ for 1 ml of blood and 1 h irradiation. The α-particle contribution to the total absorbed dose to the blood (α-dose) makes up 96.8% with an α-dose coefficient of 15.6 mGy∙kBq^−1^ while the contribution of the β-particles and conversion electrons to the total absorbed dose to the blood (β-dose) makes up only 3.2% with a β-dose coefficient of 0.5 mGy∙kBq^−1^.

For activity quantification, an average of 5.80E + 03 counts were measured for the Ra-224 emission line at 241.0 keV, 65.10E + 03 counts for the Pb-212 emission line at 238.6 keV, 3.98E + 03 counts for the Pb-212 emission line at 300.1 keV, and 3.95E + 03 counts for the Bi-212 emission line at 727.3 keV.

For the first test series (TP1), we noted that the equilibrium between Ra-224 and its progeny was not reached when we conducted the experiment. For the other two test series (TP2 and TP3), our activity measurements showed that there was equilibrium between Ra-224 and its progeny.

At the time when the Ra-224 solution was added, the activity concentration in the blood samples ranged between 0.61 and 7.90 kBq∙ml^−1^.

### α-tracks and their dependency to the absorbed dose to the blood

In 18 samples with α-doses ranging from 0 to 123 mGy, the number of α-tracks in 100 cells was enumerated as described previously [19]. The non-irradiated baseline samples did not display any α-tracks, whereas all irradiated samples contained a minimum of 3 α-tracks and a maximum of 29 α-tracks per 100 cells.

**Fig. 2:**
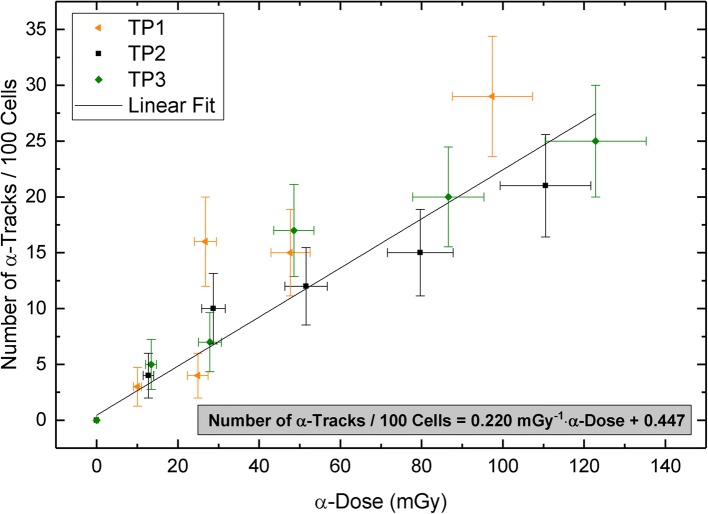
Number of α-tracks per 100 cells as a function of the α-dose. The data points of all three test persons are shown, but only the pooled data points of TP2 and TP3 were considered for the linear fit. The error bars along the *X*-axis denote the uncertainties of the α-dose calculation and were calculated by performing a propagation of uncertainties. The error bars along the *Y*-axis denote the standard deviation of each track per cell value assuming a Poisson distribution

To create an ex-vivo calibration curve for Ra-224, the data points of TP2 and TP3 were pooled and a linear fit (*r* = 0.961) was performed (Fig. 2). The data points of TP1 had to be excluded from this analysis, since we found that there was no equilibrium between Ra-224 and its progeny during the first test series, obstructing a reliable calculation of the absorbed doses. Nonetheless, these data points are shown (in orange) in Fig. 2 for comparison.

We obtained a calibration curve for Ra-224 with the linear equation:

$$ \mathrm{Number}\ \mathrm{of}\ \upalpha -\mathrm{tracks}\ \mathrm{per}\ 100\ \mathrm{cells}=\left(0.220\pm 0.024\right)\ {\mathrm{mGy}}^{-1}\bullet \upalpha -\mathrm{dose}+\left(0.447\pm 0.657\right) $$

Since this slope value is equal to the slope value obtained for our calibration curve for Ra-223 ((0.222 ± 0.014) mGy^− 1^), we conclude that the α-track frequency is not dependent on which α-emitter is used and that a combined ex-vivo calibration curve can be established for the two radium isotopes.

**Fig. 3:**
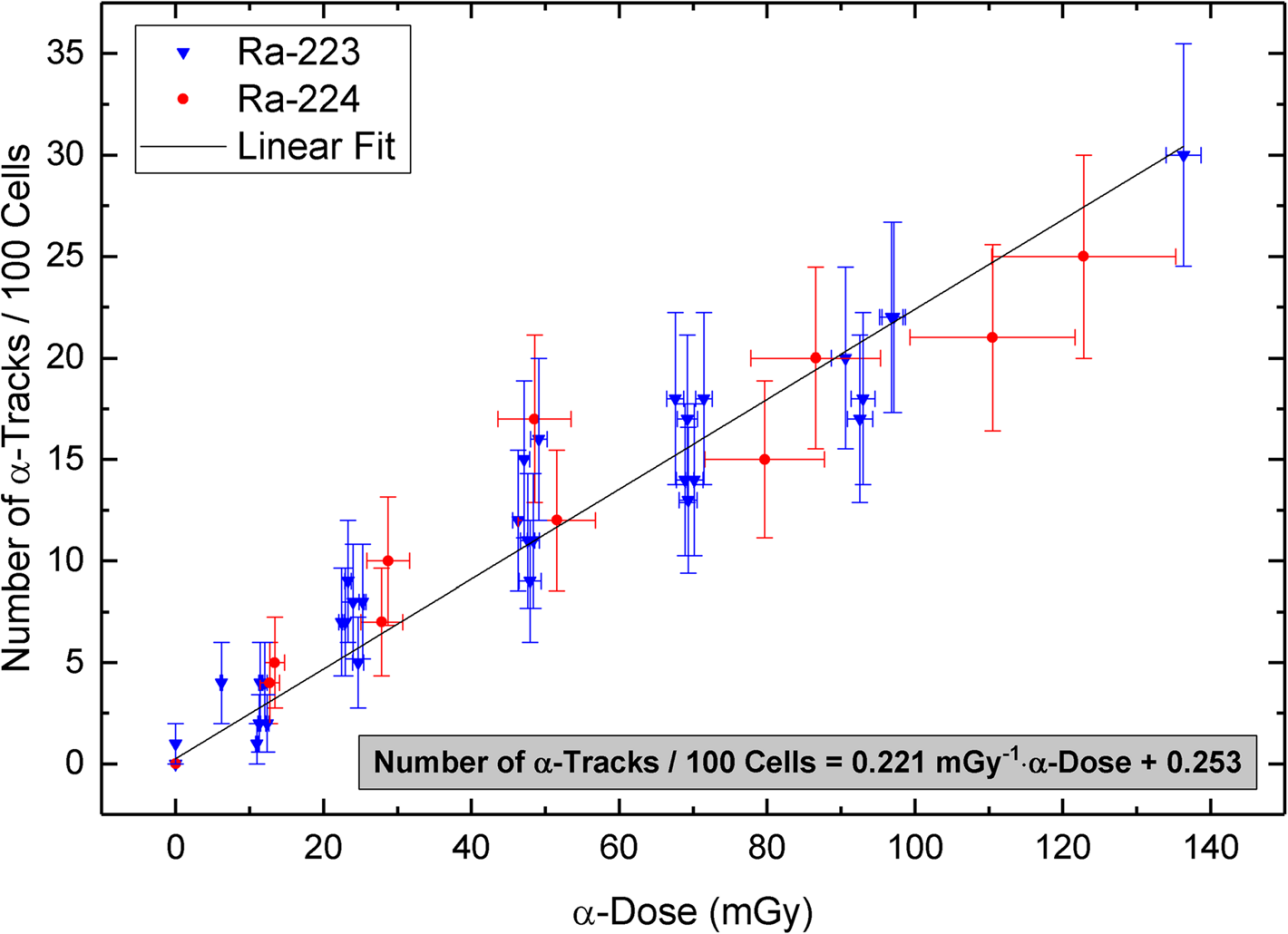
Number of α-tracks per 100 cells after irradiation with Ra-223 (blue triangles; samples from six test persons; data taken from [19]) and Ra-224 (red dots; TP2 and TP3 only) as a function of the α-dose. The straight line represents a linear fit to the pooled data

Figure 3 shows the number of α-tracks in 100 cells after irradiation with Ra-223 (data taken from [19]; blue triangles) and Ra-224 (TP2 and TP3 only; red dots) as a function of the α-dose. A linear fit (*r* = 0.966) to the pooled data points was performed resulting in a linear equation of the combined calibration curve:

$$ \mathrm{Number}\ \mathrm{of}\ \upalpha -\mathrm{tracks}\ \mathrm{per}\ 100\ \mathrm{cells}=\left(0.221\pm 0.012\right)\ {\mathrm{mGy}}^{-1}\bullet \upalpha -\mathrm{dose}+\left(0.253\pm 0.311\right) $$

### Evaluation of small γ-H2AX and 53BP1 foci

**Fig. 4:**
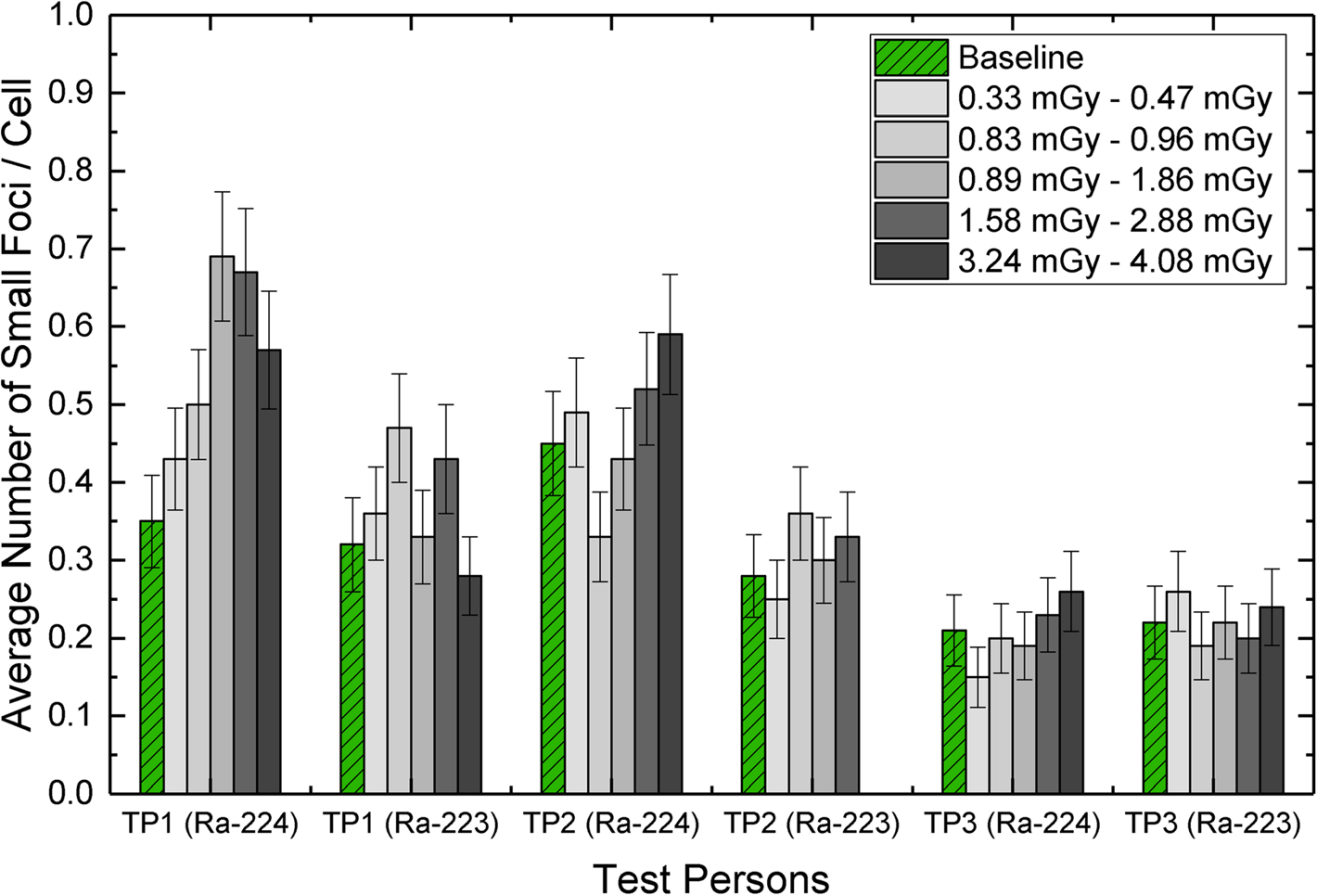
Average number of β-indicating small DSB damage foci per cell in samples irradiated with Ra-224 compared to Ra-223-irradiated samples (taken from [19]) of the same three test persons. The values for the non-irradiated baseline samples are plotted in green (with pattern); the irradiated samples are plotted in gray scale. The ranges of the corresponding β-doses are stated in the legend box to the upper left. The error bars along the *Y*-axis denote the standard deviation of each value assuming a Poisson distribution

To also evaluate the contribution of the β-dose, the number of small foci with diameters ≤ 1.1 μm was analyzed in each sample, since these represent typical low-LET-induced DSB foci [19, 24]. In accordance with the results obtained after the irradiation with Ra-223 [19], there was no correlation between the average number of small DSB-foci per cell and the β-dose after 1 h of Ra-224 irradiation. As shown in Fig. 4, the average number of small foci per cell fluctuated around the individual baseline value for each test person both after irradiation with Ra-224 and Ra-223. The large variation of foci values observed in the samples of TP1 (Ra-224) could originate from the non-existent equilibrium during the irradiation and the consequential less accurately defined absorbed doses to the blood.

## Discussion

In this work, we established a calibration curve for α-induced γ-H2AX DNA damage tracks as a function of the absorbed doses to the blood for Ra-224. Its decay properties and the total energies of the radionuclides in its decay chain deposited in ex-vivo samples of peripheral blood are comparable to those induced by Ra-223 (Ra-224: 26 MeV, Ra-223: 28 MeV). Consequently, the absorbed dose coefficients for Ra-224 (16.1 mGy∙kBq^− 1^ with an α-contribution of 96.8% and a β-contribution of 3.2%) are very similar to the absorbed dose coefficients obtained for Ra-223 (16.1 mGy∙kBq^− 1^ with an α-contribution of 96.3% and a β-contribution of 3.7%) [19].

Our ex-vivo data show that the absorbed dose-dependent number and patterns of γ-H2AX α-tracks and γ-H2AX + 53BP1 foci per nucleus are similar for Ra-223- and Ra-224-dichloride. We observed no differences in the induction of radiation-induced DNA damage structures (foci, tracks) by the two α-emitting isotopes (Figs. 3 and 4).

The limited availability of Ra-224 dichloride restricted the number of possible experiments that could be carried out. But since three of the volunteers who participated in this study were also included in our previous DNA damage study with Ra-223 [19], and given the similar decay properties of both isotopes, it appears that the results obtained with Ra-224 are depicting a valid picture of the DNA damage induced by this radionuclide.

Absorbed doses to the blood below 130 mGy were chosen for the present study as they are considered to be in a clinically relevant range. Since there are no published data for concentrations of Ra-224 in blood after clinical applications, we can only make assumptions based on the recommendations for the treatment with Ra-223. In patients treated with Ra-223, the currently recommended administered activity is 55 kBq∙kg^−1^, resulting in 4.1 MBq for an average 75 kg patient [7]. Assuming that the same amount of activity is administered when treating patients with Ra-224 and assuming a coefficient for the total absorbed dose to the blood of 4.7 mGy∙MBq^−1^, as stated in the publication of Stephan et al. [20], this results in a total absorbed dose to the blood of about 20 mGy.

For the irradiation, we chose an incubation time of 1 h, as this has proved to be suitable in previous studies and is comparable to the first sampling time point in some patient studies [19, 24–26]. Incubation times substantially longer than 1 h might lead to changes due to the progression of DNA repair and to altered blood properties [24]. Therefore, in our case, 1 h proved to be a good compromise. The decay of the radionuclides was taken into account when calculating the absorbed dose coefficients for 1 h irradiation. For our ex-vivo study, the effect of the different half-lives of Ra-223 and Ra-224 is negligible as both 11.43 and 3.63 days are long compared to the incubation time of 1 h. In a clinical setting with longer irradiation times, however, the difference in half-lives needs to be considered.

In all, the present data provide a first solid basis for further studies on the DNA damage elicited by ex-vivo experiments and in-vivo patient treatments with the radium isotopes Ra-223 or Ra-224.

## Conclusion

The correlation of the absorbed dose to the blood with the DNA damage observed in leukocytes after internal ex-vivo irradiation of blood with Ra-224 reveals values similar to an analogous Ra-223 exposure [19]. Therefore, it may be concluded that the DNA damage elicited by the two radium isotopes is comparable for the same absorbed doses. Thus, radiation-induced DNA damage-related effects in the peripheral blood are assumed to be similar after the in-vivo application of either radium isotope for a comparable absorbed dose to the blood.

## Abbreviations

AS: Ankylosing spondylitis
DSB: Double-strand break
α-dose: Contribution of α-particles to the total absorbed dose to the blood
β-dose: Contribution of β-particles and conversion electrons to the total absorbed dose to the blood
